# Noncanonical electromechanical coupling paths in cardiac hERG potassium channel

**DOI:** 10.1038/s41467-023-36730-7

**Published:** 2023-02-27

**Authors:** Carlos A. Z. Bassetto, Flavio Costa, Carlo Guardiani, Francisco Bezanilla, Alberto Giacomello

**Affiliations:** 1 Department of Biochemistry and Molecular Biology, The University of Chicago, Chicago, IL USA; 2 Dipartimento di Ingegneria Meccanica e Aerospaziale, Sapienza Università di Roma, Rome, Italy; 3 Institute for Biophysical Dynamics, The University of Chicago, Chicago, IL USA; 4 Centro Interdisciplinario de Neurociencias, Facultad de Ciencias, Universidad de Valparaiso, Valparaiso, Chile

**Keywords:** Computational biophysics, Permeation and transport, Physiology, Potassium channels

## Abstract

Voltage-gated potassium channels are involved in many physiological processes such as nerve impulse transmission, the heartbeat, and muscle contraction. However, for many of them the molecular determinants of the gating mechanism remain elusive. Here, using a combination of theoretical and experimental approaches, we address this problem focusing on the cardiac hERG potassium channel. Network analysis of molecular dynamics trajectories reveals the presence of a kinematic chain of residues that couples the voltage sensor domain to the pore domain and involves the S4/S1 and S1/S5 subunit interfaces. Mutagenesis experiments confirm the role of these residues and interfaces in the activation and inactivation mechanisms. Our findings demonstrate the presence of an electromechanical transduction path crucial for the non-domain-swapped hERG channel gating that resembles the noncanonical path identified in domain-swapped K^+^ channels.

## Abstract (web summary)

Potassium channels allow vital organ such as brain, heart, and muscles to function. Here, authors reveal the existence of a noncanonical kinematic chain of residues involving the S4/S1 and S1/S5 subunit interfaces that controls the gating of the hERG cardiac channel.

## Introduction

Voltage-gated K^+^ channels (K_V_) are membrane proteins that play key roles in many physiological processes, such as generating the nerve impulse, regulating neuronal excitability, shaping the pacemaker in the heart, and controlling muscular contractility^1^. They are organized as homotetramers of subunits that have six transmembrane segments (S1-S6). The Voltage Sensor Domain (VSD) is formed by segments S1 to S4; the Pore Domain (PD) is composed of S5, P-Loop (a membrane-reentrant region in the protein), Selectivity Filter (SF) that allows only K^+^ ions to flow through the channel, and S6. Currently, there are two tetramerized architectures identified amongst K_V_ channels: domain-swapped channels (K_V_1.2^2,3^ and K_V_7.1^4^) and non-domain-swapped channels (EAG1^5^, hERG^6^, HCN^7^, BK^8^, K_V_AP^9^, and Kat1^10^). In domain-swapped channels, the VSD from one subunit contacts the PD from the neighboring subunit whereas in non-domain-swapped channels the VSD and PD from the same subunit are in close contact.

The human ether-à-go-go–related gene^11^ (hERG or *KCNH2*) codes for a K_v_ channel involved in the delayed rectifier current^12^ (IK_r_) that determines the plateau period of the cardiac action potential. hERG malfunctions are commonly associated with severe pathologies such as long QT syndrome type 2 (LQTS-2) by loss-of-function mutations (congenital LQTS-2) or channel blockage induced by unspecific interactions with medications (acquired LQTS-2)^12–14^. These conditions have been described to promote arrhythmia and sudden death^15^.

hERG cycles over three functional states: closed, open, and inactivated. During the peak of cardiac action potential, the channels are mostly in the inactivated state; as the membrane slowly repolarizes, they rapidly recover from inactivation, thus reopening and ceasing the action potential. hERG displays a marked inward rectification, i.e., the currents carried by the channel at depolarizing potentials are relatively small compared with the currents elicited upon repolarization^12,16^. The mechanism involved in this process is due to an extremely fast pore inactivation (C-type inactivation)^17–19^, which starts before the activation process. These results suggest a possible noncanonical path that directly couples S4 movements to the C-type inactivation^20^. It has been suggested that this mechanism might depend on a structural constriction of the Selectivity Filter (SF)^21^ even if its molecular determinants remain elusive.

Recently, an exquisite feature of hERG was unveiled: the channel operates seamlessly without a covalent interaction between the voltage sensor and the pore gate^22^, strongly suggesting the presence of an alternative path that allows the channel to open excluding the S4-S5 linker loop^23,24^. Moreover, it has been shown that other regions of hERG can modulate different processes^20,25–31^ indicating that its gating mechanism is quite complex and not fully understood.

Ion channels can be viewed as allosteric machines^32^, transferring information from a sensor (VSD) to an actuator (PD). In this work, to reveal the electromechanical VSD-PD coupling mechanism in the hERG channel, we combined MD simulations to network analysis^33–35^, verifying the existence of a noncanonical route of motion that propagates across the channel. We theoretically identified a chain of residues coupling the VSD to PD that involves S4\S1 and S1\S5 subunit interfaces. Then, we validated these predictions using electrophysiological techniques revealing a path very similar to that involved in the noncanonical gating of domain-swapped channels^33,36–38^. These results provide insights into the activation and inactivation mechanisms of hERG and demonstrate the presence of a noncanonical electromechanical coupling in non-domain-swapped channels.

## Results

### Electromechanical noncanonical paths predicted by network analysis

**Fig. 1:**
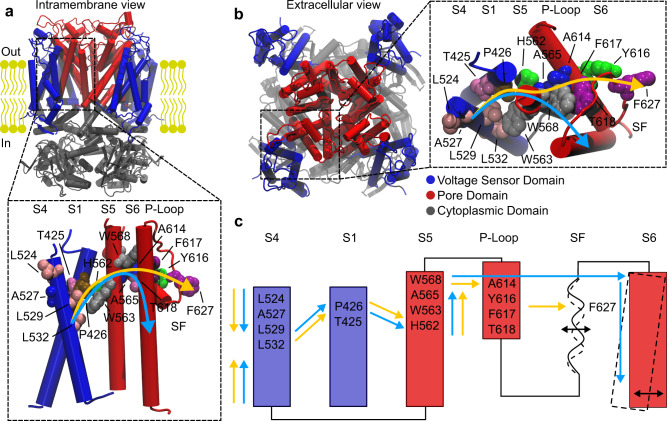
Molecular basis for the noncanonical gating paths in the hERG channel. Panels **a** and **b** show the intramembrane and extracellular views of the open state from MD simulations of the wild type^24^ colored by domains: VSD in blue, PD in red and cytoplasmic domain in gray. Panel **c** schematizes the noncanonical paths found by simulations and involved in the activation (blue arrows) and in the inactivation (yellow arrows) that couple S4-S6 and S4-SF, respectively. The residues identified using the network analysis with the highest values of betweenness centrality (see also Supplementary Table 1) that are involved in the noncanonical gating paths are indicated by a label and colored by individual amino acids using VMD 1.9.4^70^ (ResName style). The blue and yellow paths overlap until segment S5, where they diverge. To avoid confusion between the canonical and the noncanonical electromechanical coupling involved in the activation, we did not study residues on helix S6 since it would be difficult to distinguish the resulting effects on the process that depend on the canonical mechanism from those depending on the noncanonical mechanism.

Molecular Dynamics (MD) simulations of the hERG wild type (WT) in the open and closed states were run starting from the experimental structure solved by MacKinnon’s lab^6^ and the system with gating charge Q_g_ = 8e predicted in Costa et al. 2022^24^, respectively. To characterize the allosteric mechanisms of the VSD-PD coupling, the two systems were represented as networks where nodes coincide with protein residues and edges with the interactions between pairs. Each edge was assigned a weight expressed as ${w}_{{ij}}=-{{\log }}\left({C}_{{ij}}{M}_{{ij}}\right)$, which quantifies the electromechanical coupling in terms of contacts between residues and correlations of their motion, computed from MD runs. Dijkstra’s algorithm^39^ was used to determine the shortest paths between S4-S6 and S4-SF (for more details see *Methods*). Two allosteric paths were determined (Fig. 1) where the S4\S1 and S1\S5 subunit interfaces are prevalently connected by hydrophobic interactions (Supplementary Fig. 1). Analyzing the closed state trajectories, besides retrieving the canonical S4 → L45 → S6 path not discussed here but reported in Supplementary Fig. 2, we noted that displacements of the voltage sensor helix S4 propagate to helix S1. Subsequently, they pass on helix S5 and, finally, reach helix S6 in the PD determining the channel opening (blue arrows in Fig. 1). Similarly, in the open state trajectories, motion propagates from helix S4 passing through S1 and S5 following the same route as previously described for the closed trajectories. In this latter case, motion subsequently propagates to the SF via the P-Loop (yellow arrows in Fig. 1). These paths resemble the noncanonical VSD-PD coupling identified in the domain-swapped Shaker K^+^ channel ^36–38^ and they are expected to be involved in the activation and inactivation mechanisms of hERG, respectively. Interestingly, the presence of the noncanonical activation path that coexists with the classical path involving the S4-S5^23,24^ would explain the opening of the channel even if a disconnection between the VSD and the PD had been introduced^22,40^.

### Mutation of residues involved in the noncanonical paths

The role of each residue in the predicted noncanonical paths was quantified by performing betweenness centrality^41^ (BC) calculations on the wild-type systems. Residues with high values of BC (Fig. 1 and Supplementary Table 1) act as hubs in the electromechanical coupling between VSD and PD and are predominantly located on S1, S5, and the P-Loop suggesting a key role of the S1/S5 interface in the hERG noncanonical gating. Based on these theoretical predictions, the individual contribution of key residues was tested using electrophysiological assays. Residues T425, P426, A527, H562, W563, A565, W568, A614, Y616, F617, and T618 were mutated to leucine. We chose a leucine-scanning mutagenesis to perturb the identified chain and assess the role of residues in the activation and inactivation paths because the standard alanine and valine scanning approaches led to insufficient expression of the mutants. More specifically, it has been reported that alanine mutations on residues W568, Y616, F617 and valine mutations on residues A565, W568, and A614 led to channels with lack of expression^20,42^. Mutations of residues on the S4 helix (L524R, L529H, and L532H) were also produced as they had already been identified to affect the *Shaker* noncanonical gating mechanism^36^ (see the sequence alignment in Supplementary Fig. 3 between hERG (UniProt ID: Q12809) and Shaker (UniProt ID: P08510) using Clustal Omega^43^). Finally, A614 was mutated into glycine because bulkier residues such as valine^20^ or leucine (A614L—this work) did not exhibit constructs able to generate currents. Due to the lack of expression, we were not able to characterize the following residues when mutated to leucine: P426, H562, A565, and A614.

### Voltage dependence of the activation process

**Fig. 2:**
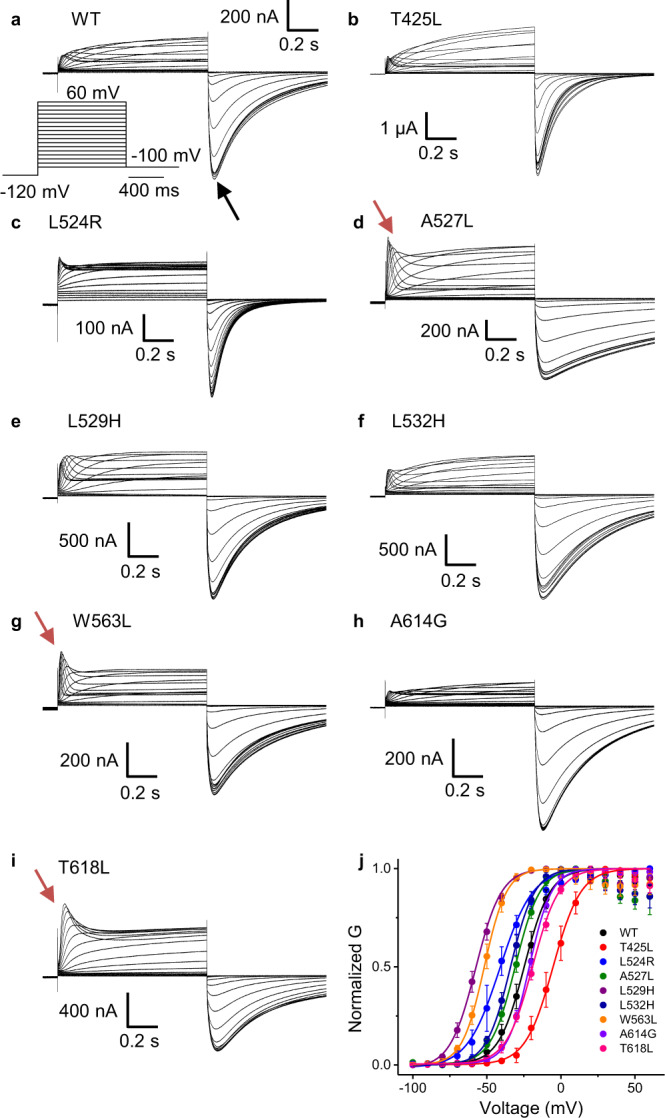
Family of current traces and conductance *versus* voltage relationship for mutants comprising the noncanonical path in hERG. **a**–**i** are the representative current traces for the WT, T425L, L524R, A527L, L529H, L532H, W563L, A614G, and T618L, respectively. Inset in a is the voltage protocol used and the black arrow denotes the time where the tail currents were measured at –100 mV to obtain the G-V curves. **j** Voltage dependence of activation (G-V curves) for the channels studied. The experimental data were fitted using Eq. 6 and the best fitted values were displayed in Table 1. The holding potential was –100 mV. Data are presented as mean ± SD (WT (*n* = 4), T425L (*n* = 4), L524R (*n* = 6), A527L (*n* = 4), L529H (*n* = 4), L532H (*n* = 4), W563L (*n* = 4), A614G (*n* = 4), and T618L (*n* = 4), where *n* is the number of biologically independent cells).

First, we set to determine whether the mutations affected the voltage dependence of the activation process. Since hERG exhibits a marked fast rectification/inactivation, the voltage dependence of activation was calculated by the relative amplitude of the peak tail currents (black arrow in Fig. 2a) plotted against the membrane potential (G-V curves). We categorized the effects of mutations on the voltage dependence of activation as left-shift and right-shift mutants (Fig. 2 and Table 1). Left-shift mutants favor the open state of the channel lowering the activation energetic barrier: they were L524R, A527L, L529H, L532H, and W563L. The magnitude of the shift in G-V varied, with the largest left shift happening in L529H and W563L, about −30 mV, whereas the smallest left shift occurred in A527L, about −6 mV (Table 1 and Fig. 2). The mutants that have a right shift favor the closed state of the channel: T425L (~20 mV); A614G (~3 mV) and T618L (~5 mV) show only a minor right shift. The time course of the current exhibited different phenotypes and three stand out. The mutants A527L (S4) (Fig. 2d), W563L (S5) (Fig. 2g) and T618L (P-Loop) (Fig. 2i) exhibited a marked peak (red arrows) with a large outward current compared to their inward tail and with the wild type channels. These results suggest that the inactivation/rectification of these channels were relieved, even though the voltage dependence of these channels were different.

**Table 1:** Fitted values for G-V and voltage-dependence of inactivation

	GV		Inactivation	
	V_G1/2_	z_G_	V_I1/2_	z_I_
WT	−24.6 ± 0.5	2.8 ± 0.1	−61.8 ± 0.7	0.9 ± 0.1
T425L	−5.2 ± 0.4	2.6 ± 0.1	−34.8 ± 1.1	0.7 ± 0.1
P426L	N. E.			
L524R	−39.6 ± 0.7	2.1 ± 0.1	−89.6 ± 1.5	0.7 ± 0.1
A527L	−30.8 ± 1.6	2.9 ± 0.5	−78.6 ± 0.6	0.9 ± 0.1
L529H	−56.7 ± 0.9	2.8 ± 0.3	−77.5 ± 1.6	0.8 ± 0.1
L532H	−33.9 ± 1.4	3.0 ± 0.4	−86.6 ± 1.5	0.6 ± 0.1
H562L	N. E.			
W563L	−51.7 ± 1.0	3.3 ± 0.4	−60.7 ± 0.7	0.9 ± 0.1
A565L	N. E.			
A614G	−21.2 ± 0.2	3.3 ± 0.1	−69.7 ± 2.0	1.1 ± 0.1
T618L	−18.9 ± 0.7	2.7 ± 0.2	−63.0 ± 0.7	0.9 ± 0.1

*N. E.* No expression mutants.

### Voltage dependence of the inactivation process

**Fig. 3:**
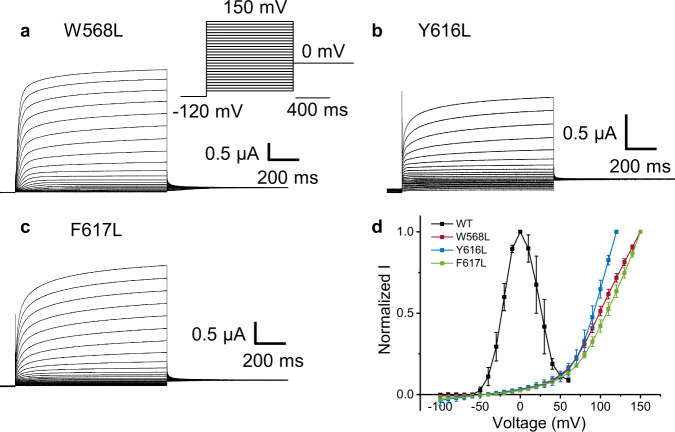
Leucine mutations at W568, Y616 and F617 abolish inactivation of hERG. **a**–**c** are the representative current traces for the W568L, Y616L, F617L, respectively. Inset in a is the voltage protocol used. For mutant Y616L the highest voltage applied was +120 mV. Because those mutants are right shifted, we had to modify the voltage protocol used in Fig. 2a to account for the shifts. **d** The peak current normalized by its maximum plotted against the membrane voltage. Data are presented as mean ± SD (WT (*n* = 3), W568L (*n* = 4), Y616L (*n* = 6), F617L (*n* = 3), where *n* is the number of biologically independent cells).

Three of the studied mutants exhibited no inactivation: W568L, Y616L, and F617L (Fig. 3). Residue W568 was previously identified to couple/interact with the selectivity of the P-loop^20^; here our network analysis showed that it is also involved in the noncanonical path, accounting for its lack of inactivation (Fig. 3a) as previously shown for W568L^20^. Similarly, mutants Y616L and F617L exhibit no inactivation, and a marked right shift in the G-V curves (Fig. 3b–d). These results suggest that these residues are involved in both hERG activation and inactivation.

**Fig. 4:**
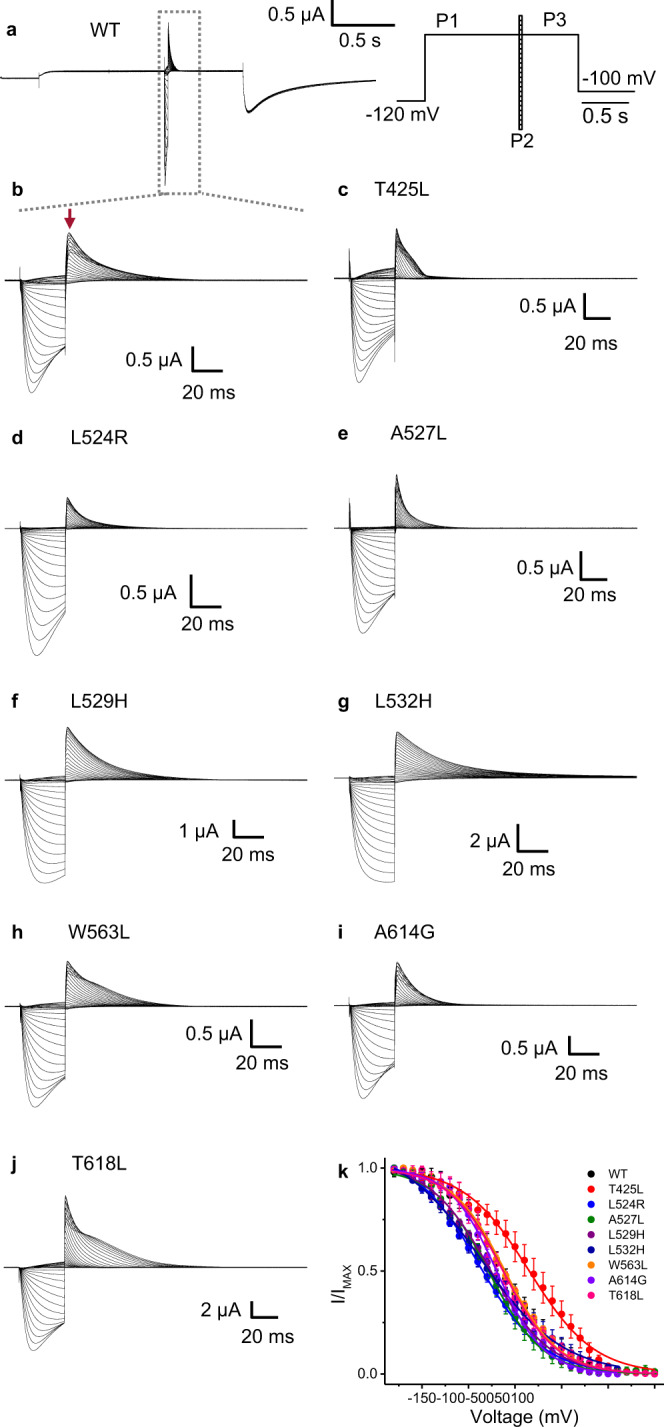
Voltage dependence of inactivation for mutations involved in the noncanonical path in hERG. **a** Currents elicited using a classical three-pulse voltage protocol shown inset in a. A voltage pulse to +20 mV (P1) was applied for 1 s to fully activate/inactivate the channel, followed by a voltage pulse (P2) applied to different potentials from −180 to +60 mV for 30 ms. This pulse was used to remove inactivation without allowing sufficient time for significant deactivation to occur. A final pulse (P3) was then applied to +20 mV for the reopening and allowed the channels to reenter into the inactivated state. For better appreciation of the currents elicited by pulses 2 and 3, we expanded the time window of the current traces (dashed grey square in **a**). **b**–**j** are, respectively, the representative expanded time window for WT, T425L, L524R, A527L, L529H, L532H, W563L, A614G, and T618L current traces. **j** Voltage dependence curve for inactivation. The voltage dependence of inactivation was assessed by the peak currents elicited by P3 (red arrow in **b** and normalized by its maximum (I/I_MAX_). For hyperpolarized potentials, <−120 mV, the channels start to deactivate which reduces the current elicited by P3, which underestimates the peak current. In order to account for this process, we corrected by extrapolating the currents back to the start of P2 from the falling phase of the currents elicited by P2^18^. For the right-shifted mutant T425L, for the mutant A527L P1 and P3 were set to +80 mV to account for the shift in the G-V curve and to fully inactivate the channel. Similarly, for T618L mutant, P1 and P3 were set to +100 mV to fully inactivate the channel. **k** Voltage dependence data for mutants. The voltage dependence data were fitted using Eq. 7 and the best-fitted values were displayed in Table 1. Data are presented as mean ± SD (WT (*n* = 4), T425L (*n* = 6), L524R (*n* = 4), A527L (*n* = 5), L529H (*n* = 3), L532H (*n* = 5), W563L (*n* = 3), A614G (*n* = 5), and T618L (*n* = 4), where *n* is the number of biologically independent cells).

Next, since the chain of residues identified via MD simulations connects the VSD to the SF that is involved in the inactivation process^21^, we hypothesized that the mutants tested here would affect the inactivation process if they were involved in a noncanonical path. As predicted, electrophysiological measurements confirm that they affect the inactivation (Fig. 4 and Table 1). L524R, A527L, L529H, L532H, and A614G showed a left shift with different magnitudes in the voltage-dependence of inactivation (Table 1). T425L showed a 27 mV right shift while W563L and T618L displayed no change in the voltage dependence of the inactivation. Interestingly, the S4 mutant L529H and the S5 mutant W563L exhibited similar behavior: the G-V curve is shifted to the left by ~−30 mV, but the voltage dependence of inactivation is left shifted only by ~−15 mV for L529H or does not have shift as for W563L. The opposite happened to the S4 mutation L532H, where the G-V curve is shifted to the left by ~−10 mV, but the inactivation is shifted to the left ~−25 mV. Another interesting result concerns the S1 mutant T425L for which the G-V is right-shifted by 20 mV, but the inactivation is right shifted by ~27 mV. The shifts in the voltage dependence of activation and inactivation were distinct regarding the amplitude and directionality, suggesting an impairment in the coupling between activation and inactivation. Therefore, they support the evidence for the noncanonical path proposed here. Since mutants W568L, Y616L, and F617L (Fig. 3) did not exhibit signs of inactivation we did not further explore the inactivation effects of these mutants.

### Comparison between theoretical predictions and experiments

**Fig. 5:**
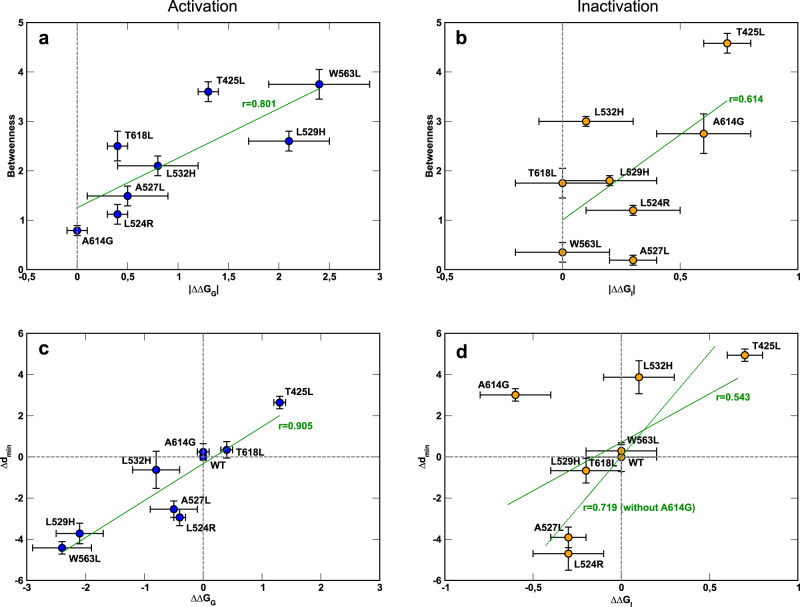
Scatter plots comparing the betweenness centrality of WT residues and the average minimal path length variation (Δd_min_ = d_minMUT_—d_minWT_, see Methods for the definition of d_min_) of mutants with respect to WT residues along the noncanonical paths against the free-energy perturbation of activation (ΔΔG_G_) and of inactivation (ΔΔG_I_). Panels **a** and **b** refer to betweenness centrality vs | ΔΔG_G_ | and betweenness centrality vs | ΔΔG_I_ | , respectively. Panels **c** and **d** refer to Δd_min_ vs ΔΔG_G_ and Δd_min_ vs ΔΔG_I_, respectively. Green lines are linear fits to the data computed by the regression analysis derived by *n* = 8 independent samples (i.e., points in each panel) and “r” is the corresponding correlation coefficient. The values of ΔΔG_G_ and ΔΔG_I_ are presented on Table 3. They were evaluated from Eqs. 8 and 9 from the fitted values obtained from Figs. 2j and 4k, respectively. The procedure to obtain ΔΔG_G_ and ΔΔG_I_ is described in Methods. Data are presented as mean values ± standard error (SE) associated to the energies and mean ± SD for betweenness centrality and the average minimal path length variation. The number of cells of biologically independent experiments associated with calculations of the activation and inactivation energies and the standard error associated to them are shown in Figs. 2j and 4k, respectively.

The theoretical predictions on the importance of residues involved in the noncanonical paths were verified by experiments as confirmed by plotting the betweenness centrality of each mutated residue along the WT noncanonical paths against the free-energy perturbation of activation (ΔΔG_G_) and of inactivation (ΔΔG_I_) (see Methods). From the plots shown in Fig. 5, it is possible to appreciate correlations between BC of the wild-type residues with |ΔΔG_G_| for activation and |ΔΔG_I_| for inactivation (Fig. 5a, b). To characterize microscopically the individual role of these residues, mutants were also produced by computational mutagenesis. We analyzed 150 ns simulations with the same network-theoretical approach. In general, the noncanonical paths involved in both activation and inactivation were not qualitatively modified. However, there were significant differences in terms of path lengths (Supplementary Table 2), which account for an exponential improvement or impairment of information transfer. Since the mutations did not substantially alter the contact patterns, these differences depend only on how effectively the motion propagates between VSD and PD. To provide a microscopic interpretation of the experimental data, we also plotted the minimal path length variation (Δd_min_) of each mutant along the noncanonical paths against ΔΔG_G_ and ΔΔG_I_ which are again correlated. These plots highlight that lower efficiency in the motion propagation (higher Δd_min_) correlates with an increasing impairment of the activation/inactivation mechanisms (higher ΔΔG). Similarly, a more efficient transfer of motion (lower Δd_min_) correlates with improved gating as measured by experiments (lower ΔΔG). These data demonstrate a fair agreement between computational and experimental results reinforcing the notion that the residues identified are part of the noncanonical electromechanical coupling in hERG.

Figure 5c shows a strong correlation between mechanical coupling and effectiveness of activation, as shown by the correlation coefficient *r* = 0.905. For inactivation, Fig. 5d shows a moderate correlation that improves if the main outlier A614G is excluded by the regression analysis. The correlation of computational and experimental data is somewhat reduced for inactivation possibly because of the smaller values of z_I_FV_I1/2_ associated to smaller variations of the gating charge z_I_ and larger relative errors on ΔΔG_I_. Since glycine is known to generate bends in the helix, we observe that the glycine replaced at A614 determines a marked bending of the P-Loop perturbing the structural arrangements and inducing a constriction of the SF (Supplementary Fig. 4). Hence, inactivation is favored in this mutant when compared to the wild type (Fig. 4j, Tables 2 and 3) even if this is not apparent in simulations, because it does not lie on the main path. Finally, simulations predict that mutants W568L, F616L, and Y617L (Supplementary Table 2) do not exhibit inactivation consistently with experimental data (Fig. 3), demonstrating the robustness of the current approach.

**Table 2:** Free-energy of activation and inactivation evaluated for WT and all the mutant channels

Energy ΔG (kcal/mol)		
	ΔG_G_ (Activation)	ΔG_I_ (Inactivation)
WT	−1.6 ± 0.1	−1.2 ± 0.1
T425L	−0.3 ± 0.1	−0.6 ± 0.1
L524R	−1.9 ± 0.1	−1.5 ± 0.2
A527L	−2.1 ± 0.4	−1.6 ± 0.1
L529H	−3.7 ± 0.4	−1.4 ± 0.2
L532H	−2.4 ± 0.4	−1.2 ± 0.2
W563L	−4.0 ± 0.5	−1.2 ± 0.1
A614G	−1.6 ± 0.1	−1.8 ± 0.1
T618L	−1.2 ± 0.1	−1.3 ± 0.2

**Table 3:** Perturbation of each mutant on the free-energy from G-V and voltage-dependence of inactivation curves

Energy ΔΔG (kcal/mol)		
	ΔΔG_G_ (Activation)	ΔΔG_I_ Inactivation
T425L	1.3 ± 0.1	0.7 ± 0.1
L524R	−0.4 ± 0.1	−0.3 ± 0.2
A527L	−0.5 ± 0.4	−0.3 ± 0.1
L529H	−2.1 ± 0.4	−0.2 ± 0.2
L532H	−0.8 ± 0.4	0.1 ± 0.2
W563L	−2.4 ± 0.5	0.0 ± 0.2
A614G	0.0 ± 0.1	−0.6 ± 0.2
T618L	0.4 ± 0.1	0.0 ± 0.2

## Discussion

Several recent works have highlighted the presence of a new mechanism that couples the VSD to the PD excluding the S4–S5 linker in the domain-swapped Shaker channels^36–38^. This alternative path was defined as “noncanonical” because it extends beyond the classical electromechanical coupling based on the action of the S4–S5 linker. Recently, computational studies have shown the presence of a similar alternative path in the hERG channel, suggesting that a noncanonical activation mechanism can also play a role in non-domain-swapped channels^24^. Here, we combined theoretical and experimental approaches to characterize this noncanonical electromechanical coupling mechanism in the hERG channel.

First, using MD simulations combined with a network analysis, we identified two paths, not related to the S4-S5 linker, that couple the VSD-PD (activation) and VSD-SF (inactivation), where the S4\S1 and S1\S5 subunit interfaces are the connecting points between VSD and PD. Interestingly, the S4/S5 interface was shown to play a role also in domain-swapped K^+^ channel^36–38^. For the activation path, simulations of the closed system with Q_g_ = 8e produced in Costa et al. 2022^24^ were analyzed and a noncanonical path that follows the S4 → S1 → S5 → S6 route was found. The choice to predict the activation path on the closed state of hERG relies on a large body of experimental evidence suggesting that the open state is intrinsically more stable than the closed state^44–47^. It has been shown that the VSD must exert work to close the pore^44,48–50^ and, when no electric field is applied, the channel “falls” into the open state^51^. These results suggest that the noncanonical communication paths for activation are not present in the open state, and they gradually build as the channel approaches the closed state. Determining the weights of the protein network from simulations of the closed state, therefore, seems the best strategy to capture the features of a fully developed communication network. Despite the uncertainty on the structure of the closed state, which was generated via homology modelling and steered MD simulations, the high correlation in Fig. 5c suggests that the noncanonical communication path for the activation revealed in this work may actually be the underlying mechanism that explains the seamless function of the channel without a covalent interaction between the voltage sensor and the pore gate^22,40^. It is important to stress that the representation of the hERG channel employed in our work may be a simplified model of the system. For instance, while we consider a single closed state, in Markov State models, hERG requires a representation with three closed states, an open state, and an inactivated state^52^. Within this framework hERG gating could be much more complex, possibly including different pathways for the transition between the different closed states and the open state. Even if this scenario is fascinating, the unavailability of experimental structures for the three closed states currently precludes computational characterization using our MD/network approach. The consistency between computational predictions and experiments, however, suggests that our model, though possibly simplified, captures the essential features of the hERG gating mechanism.

The network weights for the computation of the inactivation paths were derived from equilibrium simulations of the open state because the structure of hERG inactivated state is currently rather elusive. Even though a recent computational work^21^ has suggested a mechanism of constriction of the selectivity filter, more experimental data are still necessary to fully elucidate this mechanism. Moreover, it seems that the structure of the inactivated state may be very similar to that of the open state, consistently with the extremely fast open-to-inactivated transition^6^. Given the current lack of structural information, we decided that using the open state to predict the inactivation path was the safest approach. In this case, we identified a kinematic chain of residues that electromechanically propagates the movements of VSD and the constriction of the SF following the S4 → S1 → S5 → P-Loop→SF route relevant for the C-type inactivation.

The importance of residues in the electromechanical couplings was quantified by computing the betweenness centrality of each residue. As will be shown in the following, this a priori analysis of the wild type is a convenient and reliable way of guiding experimental and computational mutagenesis. We showed in Fig. 1 that residues P426 and T425 on helix S1 were identified to play a key role in the noncanonical activation and inactivation paths. These results agree with the work by Phan et al.^29^ where it has been shown that P426A and T425A perturbed the voltage dependence and the kinetics of the activation and the recovery from the inactivation. This evidence suggests that mutations of S1 residues break the connections between the VSD and the PD confirming the crucial role of helix S1 in coupling the VSD to the PD and completing the view proposed by Wang et al.^28^ of how the motions of helices S4 and S5 are connected. Similarly, helix S5 mediates the activation and the inactivation connecting helix S1 to helix S6 via W568, A565, W563, and H562. These results confirm the role of helix S5 on the hERG gating, as already shown in previous works^20,42,53^.

To confirm the theoretical predictions on the wild type, mutants were computationally and experimentally produced using leucine-scanning mutagenesis or structurally related mutations that have already been shown to be involved in the noncanonical path in Shaker channels^36^. In the corresponding simulations of the mutants, the communication paths were surprisingly similar to those of the wild-type channel, but with significantly different efficiencies (path lengths) in connecting the VSD to the PD. For activation, W563L, L529H, A527L, and L524R had a path length smaller than the wild type suggesting that the mutations increase exponentially the efficiency of the VSD-PD coupling path. Conversely, A614G, T618L, and T425L showed a weaker electromechanical coupling because they display longer path lengths. For inactivation, in L524R and A527L there were more efficient paths while in W563L, T618L, L529H, L532H, and T425L the inactivation path was less efficient. To provide unequivocal evidence of the noncanonical path we measured from experimental curves the free-energy perturbation of each channel for the activation and inactivation and related them with the change in the path length in the corresponding mutants. Our analysis was based on the correlation between the betweenness centrality vs |ΔΔG_I_| and |ΔΔG_G_|, and Δd_min_ vs ΔΔG_I_ and ΔΔG_G_, rather than establishing a qualitative threshold for the ΔΔG_I_ or ΔΔG_G_, for instance <1 kcal/mol. The reason for that is that the inactivation process has less voltage sensitivity (z_I_) when compared to the activation curves (z_G_ ~ 3 and z_I_ ~ 1), which provides lower values for ΔΔG_I_ compared to ΔΔG_G_ for the same shift in the voltage dependence (V_1/2_). Therefore, we reasoned that a correlation as that shown in Fig. 5 would provide a broader assessment of the contribution of each residue in the motion propagation through the noncanonical path and a quantification of the agreement between theoretical and experimental data. In this context, the correlations presented in Fig. 5 show that the experimental and theoretical results are in good agreement. The main exception is represented by the outlier A614G whose behavior is likely to depend on the presence of indirect effects on the structure of the protein backbone that could not be detected by the network analysis.

Interestingly, residues with high betweenness centrality in simulations, a measure of their relative importance in the path, affect prominently the gating mechanism also in experiments. Indeed, the scatter plots in Fig. 5a–d reveal that the greater the betweenness centrality values the higher the absolute values of ΔΔG for activation and inactivation. The mutagenesis study shows that residues in the noncanonical path act as hubs in the communication network affecting the activation and inactivation mechanisms. There is a clear advantage of using MD simulations and network analysis to make predictions on the paths and on the individual role of each residue. Information on the wild-type system based on BC represents a sufficient but not necessary condition for a residue to affect the gating mechanism. Thus, a residue with high BC value most likely will affect that mechanism. The main limitation of this computational tool is that it cannot guarantee that mutations on residues showing low BC values will not have an impact on activation or inactivation due to a plethora of indirect effects (e.g., A614G discussed in the Results). In this context, the correct way to use our computational results is to significantly narrow down the search and guide experimental mutagenesis.

Altogether, our work demonstrates the presence of an alternative allosteric gating mechanism in the hERG channel that involves the S4\S1 and S1\S5 subunit interfaces. The present work shows that this so-called noncanonical path plays a central role in the inactivation thus completing the picture sketched in our previous contribution^24^ where we showed that in the activation process, the noncanonical path coexists and complements the canonical one. The relative contribution of the two paths to the activation/deactivation mechanism, however, still needs a quantitative characterization through further experiments and simulations. Furthermore, these paths resemble those recently proposed for domain-swapped channels^36–38^, suggesting the presence of a common underlying mechanism of electromechanical coupling across the superfamily of K_v_ channels.

Our results shed light on the elusive activation and inactivation mechanisms of non-domain-swapped channels which might serve as a basis for studying the mechanisms leading to inherited and induced channelopathies.

## Methods

### MD simulations

The open and closed states of hERG channels were generated, as detailed in Costa et al. 2022^24^, and simulated for 500 ns in the NPT ensemble. hERG open state was produced from the experimentally solved structure (PDB ID 5VA2^6^), while the closed state was generated through homology modeling from the template EAG1 (PDB ID 5K7L^5^) and Steered MD simulations. The latter was run to pull helix S4 in the closed position corresponding to a gating charge Q_g_ = 8e, as the experimental value recorded by Zhang et al.^54^, 6.4e, increased by 20% to account for the underestimation affecting the experimental technique^55,56^. Mutants were produced from the pre-equilibrated wild-type system. At first, they were equilibrated for 6.5 ns in the NPT ensemble applying a time-varying harmonic restraint on each mutated residue and on its neighbors within a cut-off distance of 5.0 Å. The force constant, initially set to 10 kcal/mol/Å^2^, was decreased by 2 units every 0.5 ns of this simulation and then the systems were equilibrated for 150 ns in the NPT ensemble. All simulations were run with NAMD 2.14^57^ using the ff14SB force field for the protein^58^, the Lipid17 force field for the lipids^59^ and the TIP3P water model^60^. In all simulations, pressure was kept at 1.01325 bar by the Nosé-Hoover Langevin piston method^61,62^ and the temperature was maintained at 303.15 K by a Langevin thermostat with damping coefficient of 1 ps^−1^. Long-range electrostatic interactions were evaluated with the smooth PME algorithm^63^ with a grid space of 1 Å. For short-range non-bonded interactions, a cut-off of 12 Å with a switching function at 10.0 Å was used. The integration time step was 2 fs.

### Network analysis

The motion propagation at the basis of the VSD-PD and VSD-SF coupling mechanisms was studied representing the protein as a graph^64^ where nodes correspond to the protein residues and edges to the interactions between pairs. Each edge was assigned a weight expressed as:

1 $${w}_{{ij}}=-{{log }}\left({A}_{{ij}}\right)=-{{log }}\left({C}_{{ij}}{M}_{{ij}}\right)$$

here, ${C}_{{ij}}$ is a semi-binary contact map and ${M}_{{ij}}$ is a matrix that quantifies the motion correlation of residues $i,j$. As shown in Supplementary Fig. 5, ${C}_{{ij}}$ was computed with a truncated Gaussian kernel:

2 $$K\left({d}_{{ij}}\right)=\left\{\begin{array}{cc}1,&{d}_{{ij}}\le c\\ {e}^{-({d}_{{ij}}^{2}-{c}^{2})/2{\sigma}^{2}},&{d}_{{ij}} \, > \,c\end{array}\right.$$

where ${d}_{{ij}}$ is the distance between the C_α_ of the $i$ and $j$ residues and $c$ is the cut-off distance set to 7.0 Å. The width $\sigma$ of the gaussian kernel was chosen so as to attain a negligibly small value of the kernel at ${d}_{ij}=10$ Å. Specifically, we imposed $K({d}_{{cut}})={10}^{-5}$attaining $\sigma=1.48$. The contact map is computed by averaging the value of the kernel over all the frames of the trajectory:

3 $${C}_{{ij}}=\frac{1}{{N}_{{{{{\rm{frames}}}}}}}\mathop{\sum }\limits_{n=1}^{{N}_{{{{{\rm{frames}}}}}}}K({d}_{{ij}}\left(n\right))$$

The mutual information ${M}_{{ij}}$ of two random variables is a measure of their reciprocal independence or coupling and was used to quantify the motion correlation of two residues. Defining ${d}_{i}$ and ${d}_{j}$ as the displacement of the center of mass of the side chain with respect to its average position, the mutual information is:

4 $${M}_{{ij}}=\mathop{\sum}\limits_{{d}_{i}}\mathop{\sum}\limits_{{d}_{j}}{P\left({d}_{i},{d}_{j}\right)}{{log}}\frac{P({d}_{i},{d}_{j})}{P({d}_{i})P({d}_{j})}$$

and it quantifies the loss of uncertainty on the position of residue $i$ knowing the position of residue $j$. We used normalized mutual information ${{M}\prime}_{{ij}}=\frac{{M}_{{ij}}}{{H}_{{ij}}}$ where ${H}_{{ij}}$ represents the Shannon entropy^65^ of the variables ${d}_{i}$ and ${d}_{j}$. Since both the normalized mutual information and the semi-binary map assume values in the range [0, 1] the weights ${w}_{{ij}}$ will be non-negative, satisfying a requirement of Dijkstra’s algorithm^39^ that we used to compute the minimal paths between key regions centered on S4 and S6 helices and on the SF. Residues belonging to the key regions are reported in Supplementary Table 3. The d_min_ values correspond to the lowest values computed from Eq. 1. The betweenness centrality of each residue was computed with Brandes’s algorithm^41^ as implemented in the NetworkX 3.0 library^66^. A step-by-step example of network analysis is described in Supplementary Information, section “Step-by-step example of network analysis*”*.

### Statistics and reproducibility

The variability of the network weights ${w}_{{ij}}$ was determined using a block analysis^67,68^. The simulations were split into ${N}_{B}$ sub-trajectories of 25 ns, long enough to consider the ${w}_{{ij}}$ computed in the different blocks as uncorrelated measurements. The standard deviation for each element of the matrices was computed:

5 $$\sigma \left({w}_{{ij}}\right)=\sqrt{\frac{1}{{N}_{B}\left({N}_{B}-1\right)}\mathop{\sum }\nolimits_{B=1}^{{N}_{B}}{\left({w}_{{ij}}^{B}-{\bar{w}}_{{ij}}^{B}\right)}^{2}}$$

where ${w}_{{ij}}^{B}$ is the value of the weight computed in block $B$ while ${\bar{w}}_{{ij}}^{B}$ is the average over all blocks.

### Site-directed mutagenesis

Human hERG K^+^ channel cloned into SP64 vector (kindly provided by Dr. Eduardo Perozo—University of Chicago). Mutations were performed using Quick-change II technology (Stratagene, La Jolla, CA), together with custom primers from Integrated DNA Technologies (Integrated DNA Technologies, Inc., Coralville, IA). hERG cDNA and its mutants were sequenced to ensure accurate DNA sequencing, linearized by endonuclease EcoRI (New England Biolabs, Ipswich, MA) and cleaned up with a NucleoSpin Gel and PCR Clean-up kit (Macherey-Nagel, Bethlehem, PA). In vitro transcription kits were used to transcribe cDNA and generate cRNA (SP6 RNA expression kit; Ambion Invitrogen, Thermo Fisher Scientific, Waltham, MA).

### Oocytes preparation

Oocytes were harvested from *Xenopus laevis* in accordance with experimental protocols #71475 approved by the University of Chicago Institutional Animal Care and Use Committee (IACUC). The follicular membrane was digested using collagenase type 2 (Worthington Biochemical Corporation, Lakewood, NJ) – 2 mg/ml and supplemented by bovine serum albumin (BSA). Following the follicular membrane digestion, oocytes were incubated in standard oocytes solution (SOS) containing, in mM: 96 NaCl, 2 KCl, 1.8 CaCl_2_, 1 MgCl_2_, 0.1 EDTA, 10 HEPES and pH set to 7.4 with NaOH. SOS was supplemented with 50 µg/ml gentamycin to avoid contamination during incubation. After 6 to 24 hours of harvesting, defolliculated oocytes stage V-VI, were injected with cRNA (5 to 100 ng diluted in 50 nl of RNAse free water) and incubated at 18 °C prior to recording. Unless otherwise stated chemicals were purchased from Sigma-Aldrich (St. Louis, MO).

### Electrophysiological recordings

Ionic currents were recorded using cut-open oocyte voltage-clamp (COVC) method^69^. Currents were acquired by a setup comprising a Dagan CA-1B amplifier (Dagan, Minneapolis, MN) with a built-in low pass 4-pole Bessel filter. A 16-bit A/D converter (USB-1604, Measurement Computing, Norton, MA) was used by acquisition and controlled by an in-house software (GPatch64MC). Data was sampled at 1 MHz, digitally filtered at Nyquist frequency and decimated for the desired acquisition rate. Capacitive transient currents were compensated using a dedicated circuit. The voltage-measuring pipette was pulled using a horizontal puller (P-87 Model- Sutter Instruments, Novato, CA). For ionic currents measurements, the external solution was composed by (mM): KOH 12, CaOH_2_, HEPES 10, EDTA 0.1, NMDG (N-Methyl-D-glucamine) 108, pH 7.4 (adjusted with MES—Methanesulfonic acid). The internal solution was composed of (mM): KOH 120, EGTA 2, HEPES 10, pH7.4 (adjusted with MES—Methanesulfonic acid). The holding potential was set to −100 mV. Recordings were performed at room temperature (~ 17–18 °C).

### Data analysis

The voltage dependence of the channel conductance was taken by tail currents. After a long depolarizing pulse to different membrane potentials (P1), the membrane was set to a hyperpolarized voltage of −100 mV (P2). The currents elicited by P2 were normalized by its maximum of each experiment, averaged, and plotted against the membrane voltage of pulse P1 in order to obtain a conductance-voltage (G-V) curve. The G-V curves were fitted using a two-state model with the following equation:

6 $$G\left({V}_{m}\right)=\frac{1}{1+{{\exp }}\left(\frac{{z}_{G}F}{{RT}}\left({V}_{G1/2}-{V}_{m}\right)\right)},$$

where ${z}_{G}$ is the apparent valence of the charge times the fraction of the electric field of the transition and ${V}_{G1/2}$ is the voltage for 50% of the maximal conductance.

The steady-state inactivation currents were assessed using a triple-pulse protocol: a condition depolarized pulse (P1) for 1 s, followed by short (30 ms) hyperpolarizing pulses (P2) varying from –180 mV to +60 mV or +100 mV for some mutants (increments of 10 mV) and another test pulse (P3) for 0.6 s. The peak currents elicited by P3 were corrected by extrapolation of the decay phase of the currents elicited by P2^18^. The relative peak currents (I/I_MAX_) were plotted against the voltage of pulses P2. The inactivation voltage dependence was assessed by the I/I_MAX_ curves for each mutant using a two-state model with the following equation:

7 $$I/{I}_{{{{{\rm{MAX}}}}}}\left({V}_{m}\right)=\frac{1}{1+{{{{{\rm{exp }}}}}}\left(-\frac{{z}_{I}F}{{RT}}\left({V}_{I1/2}-{V}_{m}\right)\right)},$$

where ${z}_{I}$ is the valence of the apparent charge times the fraction of the electric field of the transition and ${V}_{I1/2}$ is the voltage for 50% of the maximal inactivation.

To evaluate the effect of leucine on the identified noncanonical path, we calculated the perturbation energy from all the single mutants and wild type. Thus, we used the activation free-energy (${\varDelta G}_{G}$) for each channel evaluated by the ${V}_{G1/2}$ and ${z}_{G}$ from the G-V curves, using the following equation:

8 $${\varDelta G}_{G}={z}_{G}F{V}_{G1/2}.$$

Similarly, we used the same idea to calculate the perturbation energy to the inactivation free-energy (${\varDelta G}_{I}$) using the following equation:

9 $${\varDelta G}_{I}={z}_{I}F{V}_{I1/2}.$$

To calculate energetic perturbation of each mutant on the free-energy of the activation or inactivation process (${\varDelta \varDelta G}_{G}$ or ${\varDelta \varDelta G}_{I}$, respectively), we calculated the difference between the free-energy of mutant (${\varDelta G}_{{{{{\rm{Mutant}}}}}}$) from the wild type (${\varDelta G}_{{WT}}$) channels for activation or inactivation process using the equation:

10 $$\varDelta \varDelta G={\varDelta G}_{{{{{\rm{Mutant}}}}}}-{\varDelta G}_{{WT}}$$

The standard error (SE) associated to the energies of activation or inactivation $\delta \triangle \triangle G$ were calculated using the following equations:

11 $$\delta \varDelta \varDelta G=F\sqrt{{(\delta {z}_{{{{{\rm{app}}}}}}{V}_{1/2})}^{2}+{(\delta {V}_{1/2}{z}_{{{{{\rm{app}}}}}})}^{2}},$$

Matlab (The MathWorks, Inc., Natick, MA) and Origin 9.0 (Origin Lab Corporation, Northampton, MA) were used for calculating G-Vs, Q-Vs, plotting and fitting the data. Data analysis were also performed using a program written in-house (Analysis).

### Reporting summary

Further information on research design is available in the Nature Portfolio Reporting Summary linked to this article.

## Supplementary information

Supplementary Information

Reporting Summary

## Data Availability

The data that support this study are available from the corresponding authors upon request. The MD trajectories that support the findings of this study are available in Zenodo with the identifiers: [10.5281/zenodo.7100860], [10.5281/zenodo.7371824] and [10.5281/zenodo.7372042]. The contact maps are available in Zenodo with the identifier: [10.5281/zenodo.7470218]. Accession codes of the structures used to produce the open and closed states, respectively: hERG open state PDB ID 5VA2 and EAG1 closed state PDB ID 5K7L. Accession codes used for the sequence alignment: hERG UniProt ID Q12809 and Shaker UniProt ID P08510. The data underling Figs. 2–5 are provided as Source Data File. Source data are provided with this paper.

## Source data

Source Data
