# Supplementary material for: Noncanonical electromechanical coupling paths in cardiac hERG potassium channel
Source: Nat Commun. 2023 Feb 27;14:1110. doi: 10.1038/s41467-023-36730-7 (PMC9971164; doi:10.1038/s41467-023-36730-7)
Supplement: Supplementary file 1 — Supplementary Information [file 41467_2023_36730_MOESM1_ESM.pdf]

Noncanonical electromechanical coupling paths in cardiac hERG potassium channel

SUPPLEMENTARY INFORMATION

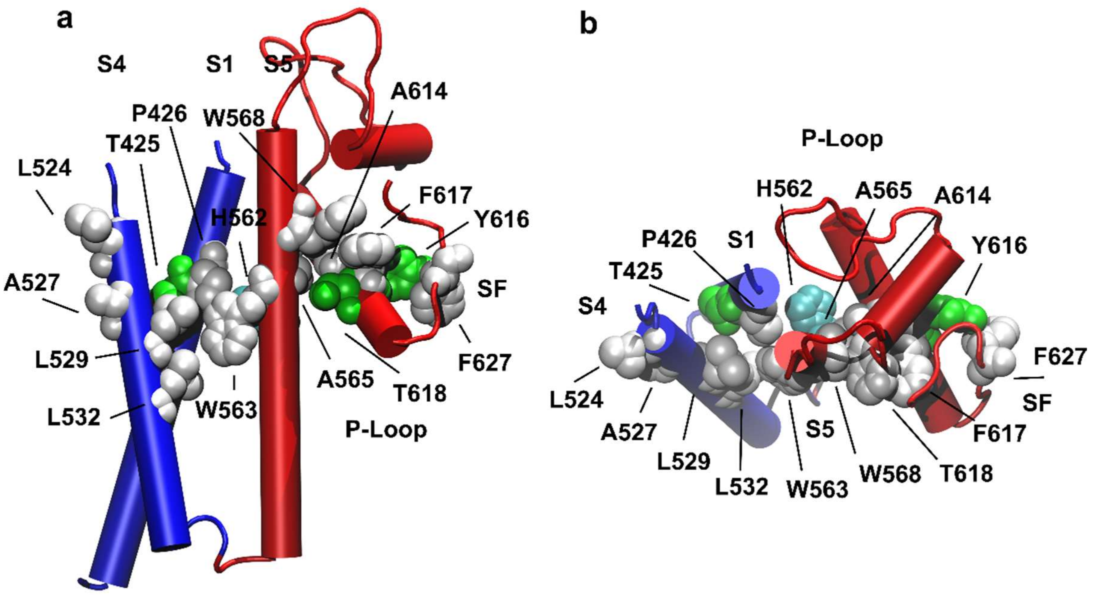

Supplementary Figure 1. Residues implicated in the noncanonical gating paths colored by individual amino acids using VMD[41] (ResType style). Panels **a** and **b** show the intramembrane and extracellular views of the open state from MD simulations of the wild type[24] colored by domains: VSD (S4 and S1) in blue and PD (S5, P-Loop, SF) in red. Non-polar residues are colored in white while polar residues are in green.

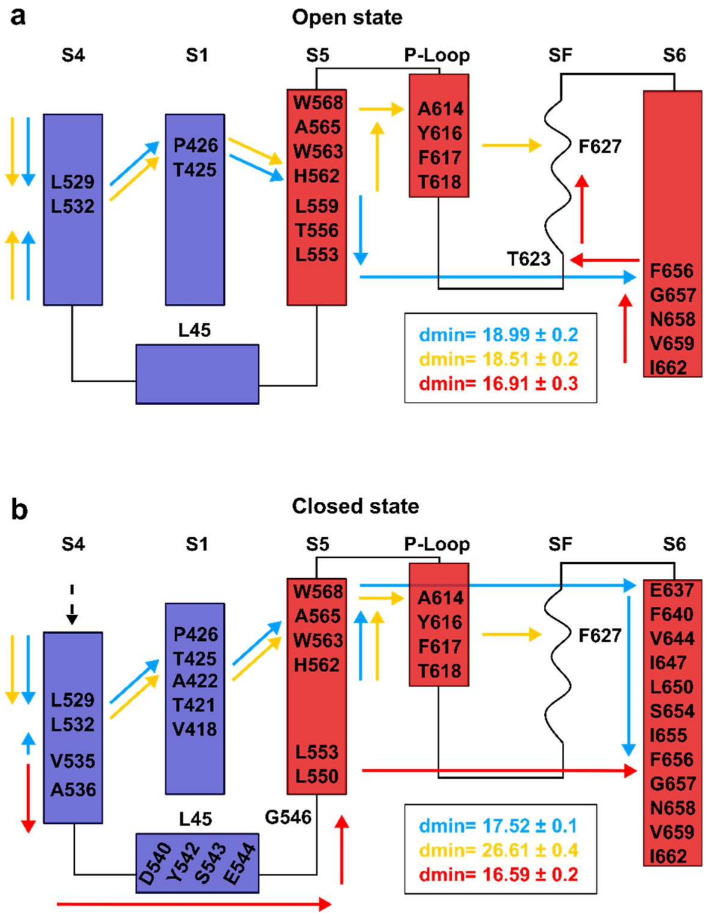

Supplementary Figure 2. Schematics of all paths identified by network analysis from the open and closed state trajectories of the WT. In panel **a**, yellow arrows refer to S4-SF coupling (noncanonical inactivation path), blue arrows to S4-S6 coupling, and red arrows to S6-SF coupling mechanism. In panel **b**, yellow arrows refer to S4-SF coupling, blue arrows to S4-S6 coupling (noncanonical activation path), and red arrows to S4-S6 coupling (canonical activation path, not discussed here, please refer to Costa et al. 2022). The arrow's direction identifies the source and sink regions used to compute the paths. The reported residues have a betweenness centrality value greater than 2.0 regarding the specific path where the residues sit on. The canonical activation path can be appreciated in panel **b** with a length of d_{min}=16.59±0.2. The inactivation path can be found also analyzing the trajectory of the closed state. However, the corresponding length of this path is higher than that computed from the open state since it reaches a value of d_{min}=26.61±0.4 instead of d_{min}=18.51±0.2. Considering the logarithmic nature of the metric used in the network analysis, a difference of eight units in d_{min} corresponds to a difference of three orders of magnitude in terms of coupling efficiency suggesting that in this system the S4-SF coupling is extremely weak. This is the reason why, as already discussed in the main text, we predicted the activation path from the closed state and inactivation path from the open state.

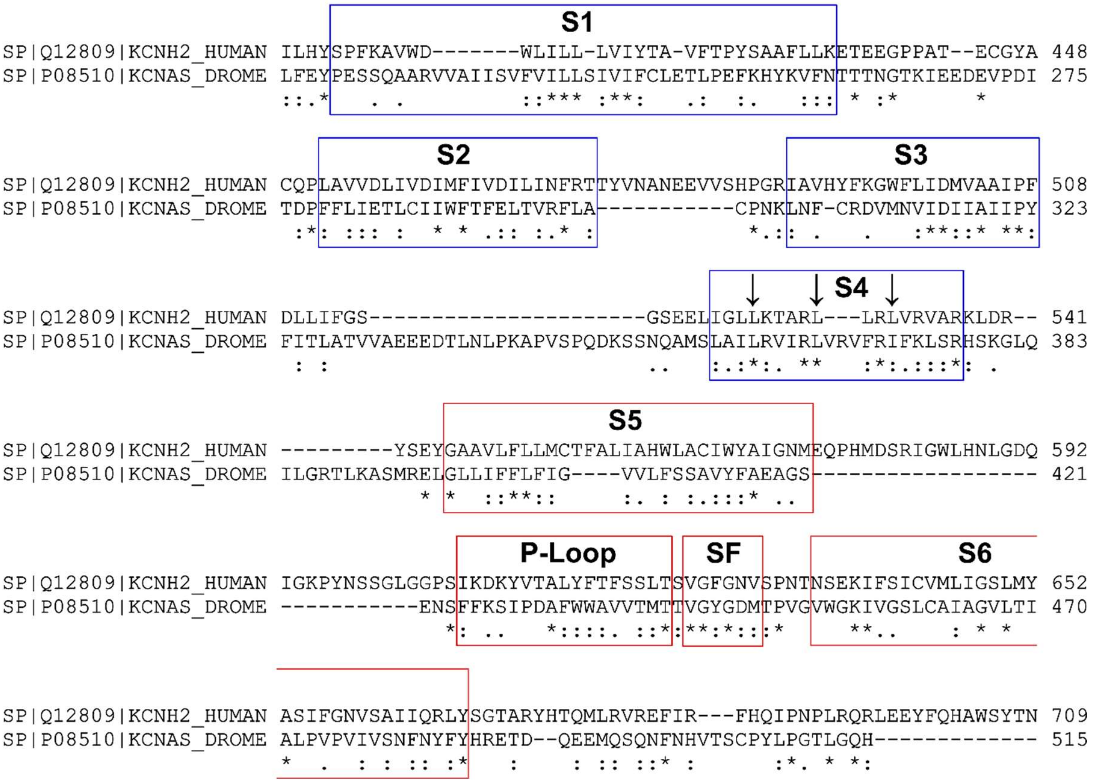

Supplementary Figure 3. Sequence alignment between hERG (UniProt ID: Q12809) and Shaker (UniProt ID: P08510) using Clustal Omega[44]. Boxes refer to helices of the VSD (in blue) and of the PD (in red). Arrows indicate the hERG residues L524, L529, and L532.

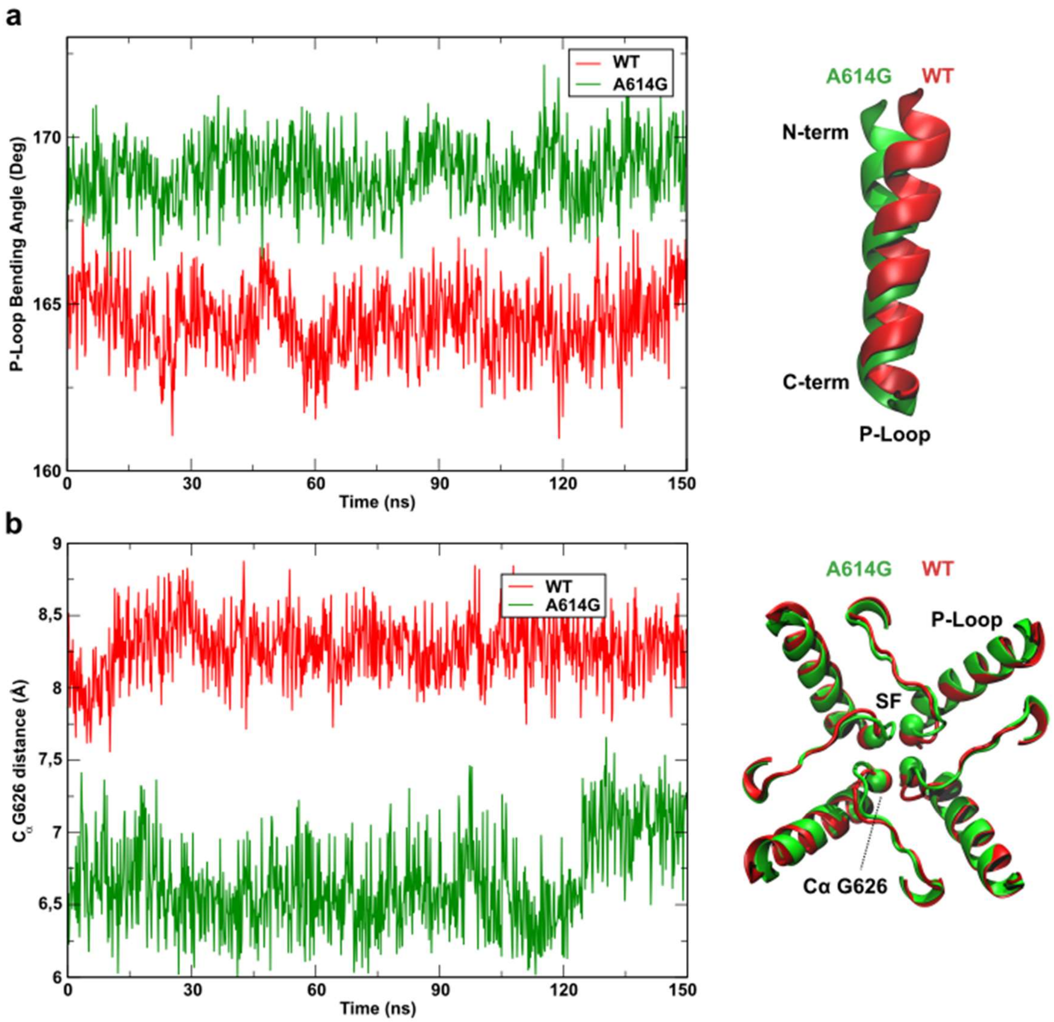

Supplementary Figure 4. Asymmetrical constriction of the SF induced by A614G mutation. Panel **a** shows the bending angle of the P-Loop computed as $\theta = \arccos \frac{\vec{v_{CT}}\vec{v_{CB}}}{|\vec{v_{CT}}||\vec{v_{CB}}|}$ where $\vec{v_{CT}}$ is a vector pointing from the middle region of the P-Loop (residues 613-618) to the top of the same helix (residues 607-612) and $\vec{v_{CB}}$ is a vector pointing from the middle (residues 613-618) to the bottom end (residues 619-624). Panel **b** shows the constriction of the SF reporting the mean of the two cross-subunit distances between the C_α atoms of G626 of diagonally opposed subunits in the wild type and mutant simulations.

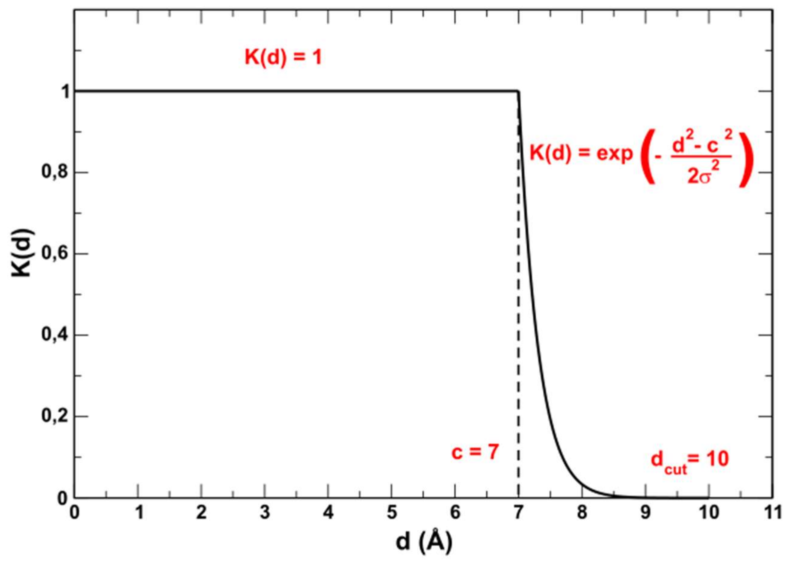

Supplementary Figure 5. The Gaussian kernel used in the definition of semi-binary contact maps.

Supplementary Table 1: Betweenness centrality values of each residue involved in the noncanonical activation and inactivation paths averaged for the four subunits. Errors are expressed as standard deviations. Residues not reported here are assumed to have BC value of 0 for the noncanonical activation and inactivation paths.

Residue		Activation	Inactivation
S4	L524	1.12 ± 0.2	1.20 ± 0.1
	A527	1.49 ± 0.2	0.19 ± 0.1
	L529	2.60 ± 0.2	1.81 ± 0.1
	L530	2.45 ± 0.3	0.40 ± 0.1
	L532	2.10 ± 0.2	3.00 ± 0.1
	V535	2.53 ± 0.1	1.14 ± 0.1
	A536	3.10 ± 0.1	0.12 ± 0.1
S1	P426	8.29 ± 0.2	6.60 ± 0.4
	T425	3.60 ± 0.2	4.58 ± 0.2
	A422	4.12 ± 0.3	2.25 ± 0.2
	T421	4.05 ± 0.1	1.90 ± 0.1
	V418	3.95 ± 0.2	0.85 ± 0.1
S5	W568	5.90 ± 0.2	5.40 ± 0.2
	A565	4.00 ± 0.1	2.25 ± 0.3
	W563	3.75 ± 0.3	0.35 ± 0.2
	H562	7.30 ± 0.1	2.50 ± 0.1
P-Loop	A614	0.79 ± 0.1	2.75 ± 0.4
	Y616	3.50 ± 0.1	5.05 ± 0.2

	F617	3.50 ± 0.1	4.95 ± 0.2
	T618	2.50 ± 0.3	1.75 ± 0.3
SF	F627	0.00	7.90 ± 0.1
	G626	0.00	10.31 ± 0.2
S6	E637	2.65 ± 0.1	3.65 ± 0.3
	F640	2.25 ± 0.1	0.80 ± 0.4
	V644	3.37 ± 0.1	0.42 ± 0.2
	I647	7.31 ± 0.1	0.23 ± 0.1
	L650	14.62 ± 0.2	0.10 ± 0.1
	S654	8.00 ± 0.3	0.01 ± 0.1
	I655	6.50 ± 0.2	0.00
	F656	10.15 ± 0.1	0.00
	G657	4.00 ± 0.1	0.00
	N658	8.50 ± 0.1	0.00

Supplementary Table 2: Noncanonical path lengths of mutants averaged for the four subunits. Errors are expressed as standard deviations.

Mutant	Activation	Inactivation
WT	d_{min}= 17.52 ± 0.1	d_{min}= 18.51 ± 0.2
T425L	d_{min}= 20.16 ± 0.3	d_{min}= 23.45 ± 0.3
P426L	No path	No path
L524R	d_{min}= 14.58 ± 0.4	d_{min}= 13.81 ± 0.8
A527L	d_{min}= 14.98 ± 0.4	d_{min}= 14.60 ± 0.5
L529H	d_{min}= 13.81 ± 0.5	d_{min}= 17.84 ± 0.6
L532H	d_{min}= 16.89 ± 0.9	d_{min}= 22.38 ± 0.8
H562L	d_{min}= 30.56 ± 0.8	d_{min}= 18.61 ± 0.2
W563L	d_{min}= 13.10 ± 0.3	d_{min}= 18.80 ± 0.3
A565L	d_{min}= 10.84 ± 0.5	d_{min}= 18.07 ± 0.4
W568L	d_{min}= 31.14 ± 0.1	No path
A614G	d_{min}= 17.76 ± 0.4	d_{min}= 21.52 ± 0.3
Y616L	d_{min}= 16.18 ± 0.4	No path
F617L	d_{min}= 17.01 ± 0.7	d_{min}= 32.11 ± 1.2
T618L	d_{min}= 17.86 ± 0.7	d_{min}= 18.50 ± 0.7

Supplementary Table 3: Residues of the key regions on S4, S6, and SF used for the network analysis.

S4	K525, T526, A527, R528, L529, L530, R531, L532, V533, R534, V535, A536, R537
S6	Y652, A653, S654, I655, F656, G657, N658, V659, S660, A661, I662
SF	V625, G626, F627, G628, N629

SUPPLEMENTARY METHODS

Step-by-step example of network analysis

In order to be more specific about our protocol, in this section we provide a step-by-step example of network analysis used for the identification of the noncanonical paths. The steps are the following:

1. Computation of the semi-binary contact map

The matrix C_{ij} that represents a semi-binary contact map, is computed with a home-developed program from equilibrium simulations. Supplementary Figure 6 shows an example of a semi-binary contact map computed for the WT system in the closed state.

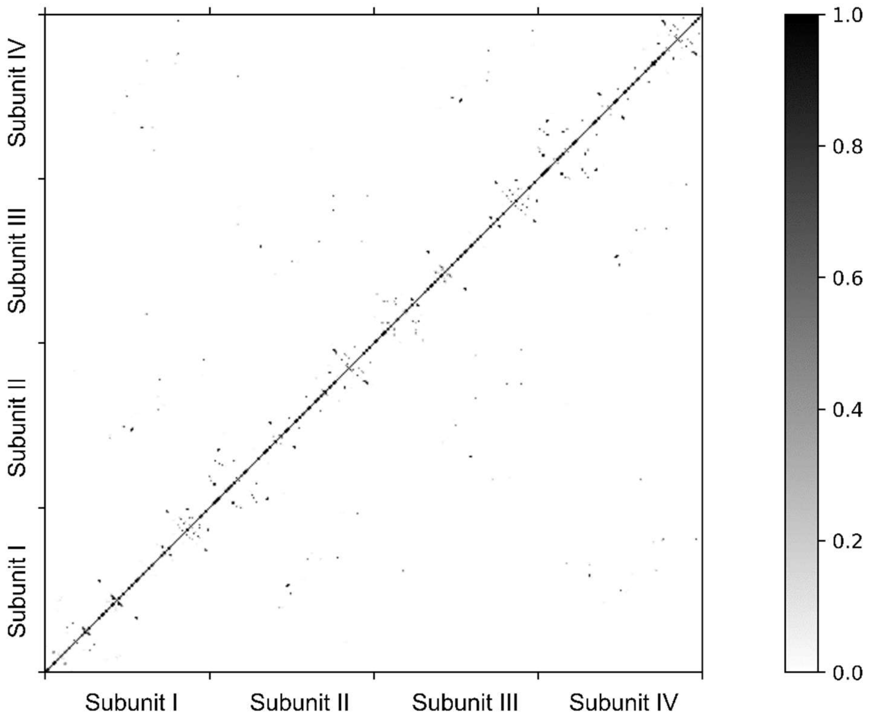

Supplementary Figure 6. Example of a semi-binary contact map computed for the WT system in the closed state with gating charge Q_g=8e. Black points correspond to interactions identified between pairs of residues.

2. Computation of the mutual information matrix.

Then, the matrix M_{ij} of the mutual information is computed using a home-developed program, as in the previous step. Supplementary Figure 7 represents the corresponding matrix computed in the WT closed state system.

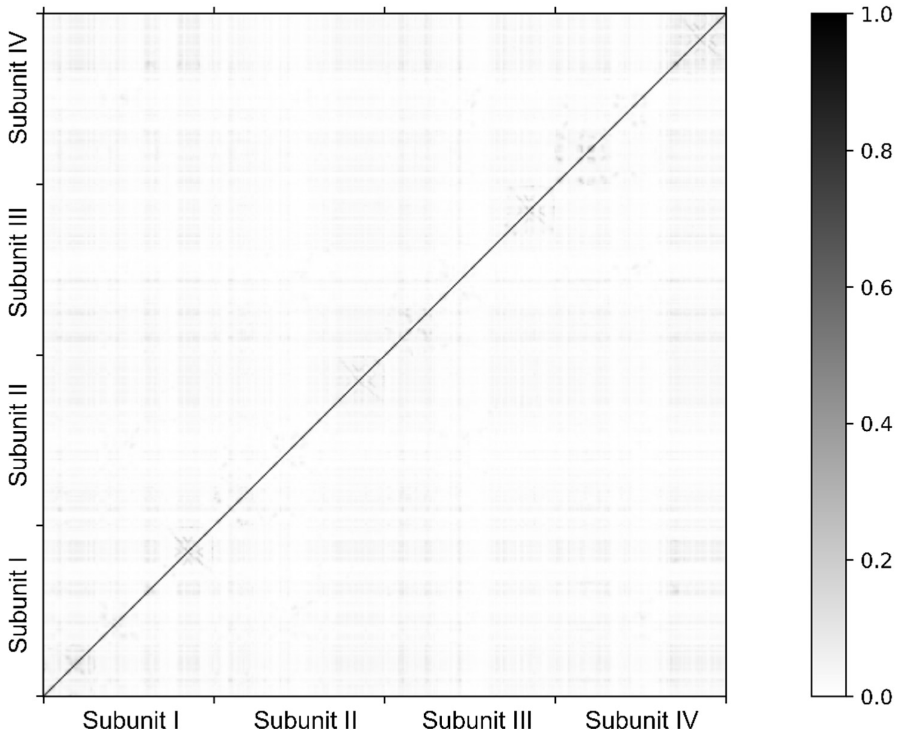

Supplementary Figure 7. Mutual information computed for the WT closed state. Black points represent pair of residues with highly correlated kinematic motion (normalized mutual information close to one).

3. Computation of the map of information distances.

The next calculation is the element-by-element product of the matrices C_{ij} and M_{ij} that is converted in the map of information distances $w_{ij} = -\log(C_{ij}M_{ij})$. The resulting data is fed to another program to perform the final path calculation. Supplementary Figure 8 shows the matrix of the information distances w_{ij}:

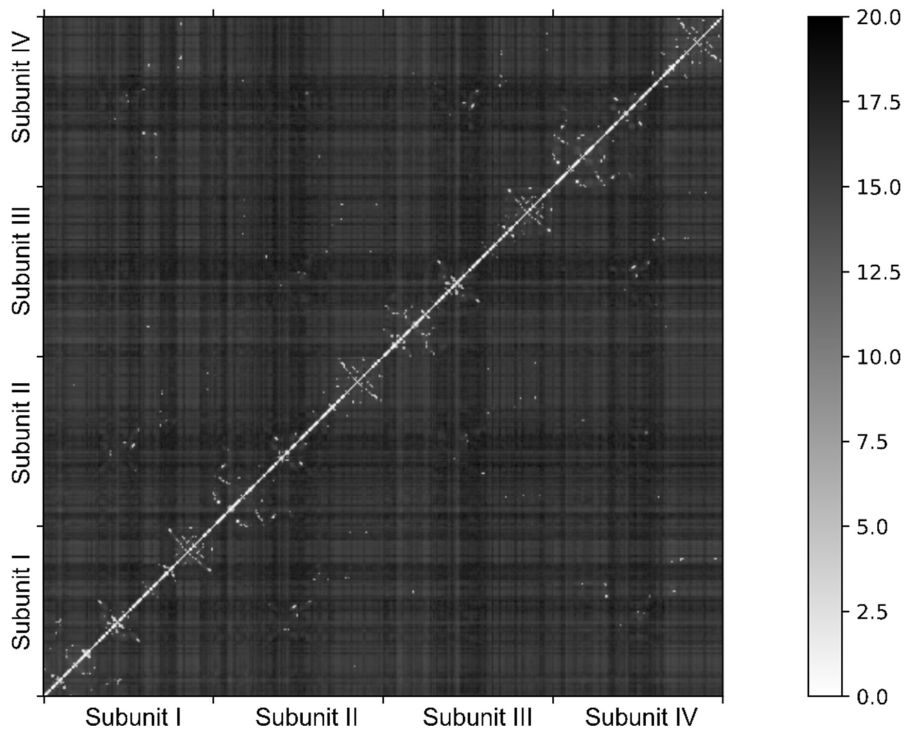

Supplementary Figure 8. Map of the metrics w_{ij}.

On the diagonal there are small values of w_{ij} since the adjacent residues on the protein sequence due to their covalent bonds have a correlated motion. Interestingly, off the diagonal there are some white points that represent connected and motion-correlated residues that are distant on the protein sequence. It must be stressed that low-weight, off-diagonal elements are the most important ones since they allow long-range communication inside the network. From the comparison of Supplementary Figures 8 with 7 and 6 it is apparent that these off-diagonal elements are much better resolved in the map of information distances w_{ij} than in the semi-binary contact map C_{ij} or in the matrix of Mutual information M_{ij}. Therefore, the information distance $w_{ij} = -\log(C_{ij}M_{ij})$ represents a better metric than C_{ij} or M_{ij} alone. The matrix of information distance is subsequently fed into another program to perform path calculation.

4. Determination of source and sink regions.

The first task performed by the program for the path calculation is the identification of the two regions, usually defined as “source” and “sink” regions, that the path connects. For the activation, key residues were chosen on helix S4 (R531) and on helix S6 (G657) to identify the source and sink regions respectively. For the inactivation, key residues were chosen on helix S4 (R531) and on the SF (F627) to identify the source and sink regions respectively. Then, the program identifies all residues spending at least 70% of the trajectory inside a

sphere centered on those key residues with a radius of 7.0 Å to encompass all residues of the helix. All residues used for the network analysis are shown in Supplementary Table 3.

5. Betweenness Centrality calculation.

Once the residues of the source and sink regions have been identified, these lists of residues (reported in Supplementary Table 3), along with the weight map, are fed into a script of the NetworkX library (https://networkx.org/) that outputs the betweenness centrality value of each residue. The higher the value of the betweenness centrality, the greater the importance of that specific residue acting as a hub in the communication network.

6. Path visualization.

Once the betweenness of all residues is known, using the VMD visualization program, we highlight on the protein structure all the residues with non-zero betweenness. The space distribution of these residues represents the communication path. As a further consistency check, minimal pathways are also computed and visualized using Dijkstra's algorithm.
