# Supplementary material for: Noncanonical electromechanical coupling paths in cardiac hERG potassium channel
Source: Nat Commun. 2023 Feb 27;14:1110. doi: 10.1038/s41467-023-36730-7 (PMC9971164; doi:10.1038/s41467-023-36730-7)
Supplement: Supplementary file 2 — Reporting Summary [file 41467_2023_36730_MOESM2_ESM.pdf]

nature portfolio

Corresponding author(s): Francisco Bezanilla and Alberto Giacomello

Last updated by author(s): Jan 27, 2023

Reporting Summary

Nature Portfolio wishes to improve the reproducibility of the work that we publish. This form provides structure for consistency and transparency in reporting. For further information on Nature Portfolio policies, see our Editorial Policies and the Editorial Policy Checklist.

Statistics

For all statistical analyses, confirm that the following items are present in the figure legend, table legend, main text, or Methods section.

n/a	Confirmed	
☐	☒	The exact sample size (*n*) for each experimental group/condition, given as a discrete number and unit of measurement
☐	☒	A statement on whether measurements were taken from distinct samples or whether the same sample was measured repeatedly
☒	☐	The statistical test(s) used AND whether they are one- or two-sided<br>*Only common tests should be described solely by name; describe more complex techniques in the Methods section.*
☒	☐	A description of all covariates tested
☒	☐	A description of any assumptions or corrections, such as tests of normality and adjustment for multiple comparisons
☐	☒	A full description of the statistical parameters including central tendency (e.g. means) or other basic estimates (e.g. regression coefficient) AND variation (e.g. standard deviation) or associated estimates of uncertainty (e.g. confidence intervals)
☒	☐	For null hypothesis testing, the test statistic (e.g. *F*, *t*, *r*) with confidence intervals, effect sizes, degrees of freedom and *P* value noted<br>*Give P values as exact values whenever suitable.*
☒	☐	For Bayesian analysis, information on the choice of priors and Markov chain Monte Carlo settings
☒	☐	For hierarchical and complex designs, identification of the appropriate level for tests and full reporting of outcomes
☒	☐	Estimates of effect sizes (e.g. Cohen's *d*, Pearson's *r*), indicating how they were calculated

Our web collection on statistics for biologists contains articles on many of the points above.

Software and code

Policy information about availability of computer code

Data collection	NAMD 2.14 (https://www.ks.uiuc.edu/Research/namd/). A in-house software (Gpatch) was used to collect the electrophysiological data.
Data analysis	VMD 1.9.4 (https://www.ks.uiuc.edu/Research/vmd/)<br>NetworkX 3.0 (https://networkx.org/)<br>Clustal Omega (https://www.ebi.ac.uk/Tools/msa/clustalo/)<br>In-house software (Analysis), Matlab (The MathWorks), and Origin 9.0 (Origin Lab) were used to analyze, plot and fit the experimental data.

For manuscripts utilizing custom algorithms or software that are central to the research but not yet described in published literature, software must be made available to editors and reviewers. We strongly encourage code deposition in a community repository (e.g. GitHub). See the Nature Portfolio guidelines for submitting code & software for further information.

Data

Policy information about availability of data

All manuscripts must include a data availability statement. This statement should provide the following information, where applicable:

- Accession codes, unique identifiers, or web links for publicly available datasets
- A description of any restrictions on data availability
- For clinical datasets or third party data, please ensure that the statement adheres to our policy

The MD trajectories that support the findings of this study are available in Zenodo with the identifiers: https://doi.org/10.5281/zenodo.7100860, https://

doi.org/10.5281/zenodo.7371824 and https://doi.org/10.5281/zenodo.7372042. The contact maps are available in Zenodo with the identifier: https://doi.org/10.5281/zenodo.7470218. Accession codes of the structures used to produce the open and closed states, respectively: hERG open state PDB ID 5VA2 (http://doi.org/10.2210/pdb5VA2/pdb) and EAG1 closed state PDB ID 5K7L (http://doi.org/10.2210/pdb5K7L/pdb). Accession codes used for the sequence alignment: hERG UniProt ID Q12809 and Shaker UniProt ID P08510. The values behind Figs. 2, 3, 4 and 5 are provided as Source Data File.

Human research participants

Policy information about studies involving human research participants and Sex and Gender in Research.

Reporting on sex and gender	N/A
Population characteristics	N/A
Recruitment	N/A
Ethics oversight	N/A

Note that full information on the approval of the study protocol must also be provided in the manuscript.

Field-specific reporting

Please select the one below that is the best fit for your research. If you are not sure, read the appropriate sections before making your selection.

☒ Life sciences ☐ Behavioural & social sciences ☐ Ecological, evolutionary & environmental sciences

For a reference copy of the document with all sections, see nature.com/documents/nr-reporting-summary-flat.pdf

Life sciences study design

All studies must disclose on these points even when the disclosure is negative.

Sample size	We did not perform statistical test to determine the sample sizes. Electrophysiological experiments were replicated from 3 to 6 independent repetitions of the same experimental procedure using different oocytes from different frogs. No repeated measurements were performed using the same oocyte.
Data exclusions	No data was excluded from analysis other than those that clearly indicated unsuccessful expression of the protein by the oocytes and leaky cells.
Replication	Different batches of oocytes were used for the heterologous expression of the channels. Reproducibility was attested by the similar biophysics features of the channels tested over different batches of oocytes from different frogs.
Randomization	Different frogs used as oocytes donors were wild type frogs were randomly selected from our colony on the day of oocytes surgical extraction without any criteria that would bias the outcome. After the digestion of the ovaries, the oocytes were randomly selected prior to injection. On the day of the experiments, they were also randomly selected prior to electrophysiological experiments.
Blinding	Blind experiments were not performed since our analysis were systematically done.

Reporting for specific materials, systems and methods

We require information from authors about some types of materials, experimental systems and methods used in many studies. Here, indicate whether each material, system or method listed is relevant to your study. If you are not sure if a list item applies to your research, read the appropriate section before selecting a response.

Materials & experimental systems

n/a	Involved in the study	
☒	☐	Antibodies
☒	☐	Eukaryotic cell lines
☒	☐	Palaeontology and archaeology
☐	☒	Animals and other organisms
☒	☐	Clinical data
☒	☐	Dual use research of concern

Methods

n/a	Involved in the study	
☒	☐	ChIP-seq
☒	☐	Flow cytometry
☒	☐	MRI-based neuroimaging

Animals and other research organisms

Policy information about studies involving animals; ARRIVE guidelines recommended for reporting animal research, and Sex and Gender in Research

Laboratory animals	Adult female Xenopus laevis older than one year old was used as oocytes donors.
Wild animals	No wild animal was used.
Reporting on sex	Female
Field-collected samples	No field-collected sample was collected.
Ethics oversight	The University of Chicago Institutional Animal Care and Use Committee have approved all protocols and procedures for oocytes harvesting #71475.

Note that full information on the approval of the study protocol must also be provided in the manuscript.
